# Cementing techniques for total knee arthroplasty in Norwegian hospitals; a questionnaire-based study

**DOI:** 10.1186/s12891-023-07040-2

**Published:** 2023-11-18

**Authors:** Michelle Khan, Jan-Erik Gjertsen, Anne Marie Fenstad, Anders Refsum, Uy Nguyen, Geir Hallan, Paul Johan Høl, Ove Furnes

**Affiliations:** 1 Department of Clinical Medicine, Faculty of Medicine, University of Bergen, Jonas Lies Vei 87, 5021 Bergen, Norway; 2 Department of Orthopaedic Surgery, Haukeland University Hospital, Bergen, Norway; 3 Orthopaedic Department, Bærum Hospital, Vestre Viken Hospital Trust, Bærum, Norway

**Keywords:** TKA, Cementing technique, Best practices, Aseptic loosening

## Abstract

**Background:**

Cementing technique in total knee arthroplasty (TKA) may influence implant survival. There is limited knowledge about the results with clinically used techniques.

The aim of this study was to investigate cementing techniques for TKA in Norwegian hospitals, to compare widely used techniques to recommendations from the literature, and to investigate variation within hospitals.

**Methods:**

A questionnaire requesting information about cementing techniques were distributed to all Norwegian orthopedic surgeons performing TKAs regularly in 2020. Data was analyzed using descriptive statistical methods.

**Results:**

We acquired 121 responses out of 257 surgeons. They were from 45 out of 56 hospitals, and at least half of the TKA surgeons from 20 hospitals, constituting 79 surgeons. All responders used pulsatile lavage. Cement application to both the tibial plateau and stem (full cementation) was practiced by 61%. Application of cement to both implant and bone was done by 70% of surgeons. Techniques to improve cement penetration were used by 86%. Only 35% of surgeons aimed to get a cement mantle thickness between 3–5 mm. Flexing the knee joint to remove excess cement was done by 82%. We found that in 55% of 20 hospitals the surgeons did not agree on the use of common guidelines in their ward.

**Conclusions:**

The majority of the responders used recommended techniques from the literature when cementing TKA. At more than half of the eligible hospitals, surgeons disagreed about their hospitals’ use of common guidelines. Focusing on developing evidence-based guidelines would be beneficial for TKA-quality.

**Supplementary Information:**

The online version contains supplementary material available at 10.1186/s12891-023-07040-2.

## Background

Total knee arthroplasty (TKA) is the most effective treatment for complicated osteoarthritis in the knee. A contemporary TKA has a survival rate above 90% over a 10-year period [1–3], and has a high rate of satisfaction among patients [4]. However, revisions do occur, and revision surgeries have a lower overall implant-survival and health-related quality of life for the patient [5].

There are multiple indications for revision, but aseptic loosening of the tibial component is the most common one. Aseptic loosening of the tibial component has not declined over a 20-year period [6, 7], making it a threat to implant survivorship despite the decline in total number of revisions. Aseptic loosening is likely associated with the surgical technique. According to Scuderi et al., the most decisive time of a TKA is the operative procedure and the outcome is influenced by the surgical technique and implant design [8].

Although cementing technique of a TKA likely plays an important role for the implant survival, no guidelines for the surgical procedure exists in Norway [9]. It is uncertain if the most optimal techniques are used. One of the main goals of the techniques is to achieve sufficient penetration of cement into cancellous bone, ensuring a strong cement-bone interlock. It has been suggested that a penetration between 3–5 mm is sufficient [10], and that cement thickness, cement from metal to deepest point into cancellous bone, is positively associated with the implant survival [11].

The aims of this study was to investigate how Norwegian orthopedic surgeons cement contemporary TKAs, and to compare their practices to techniques advised in evidence based techniques from the literature, as described in the scoping review by Refsum and Nguyen [9]. Further, we wanted to investigate if hospitals follow common guidelines for cementing TKAs.

## Methods

A questionnaire was designed to obtain information via Google Forms (Supplementary table 1, see Supplementary information). The questionnaire constituted 34 questions; 5 questions addressing hospital affiliation and surgeon experience, the hospitals use of guidelines for cementing technique. The remaining 29 questions acquired information about cementing technique, such as bone preparation, cement preparation—and application of cement on bone and implant, implant insertion and curing position. The majority of questions were multiple choice where the surgeons selected the most fitting alternative. Free text questions were designed where further elaboration was needed. Mailing lists from the Norwegian Orthopedic Association and a closed Facebook-group for working orthopedic surgeons in Norway were the platforms used to distribute the questionnaire. A reminder was distributed 4 weeks after primary distribution. An inquiry was sent by mail to the Norwegian Arthroplasty Registers (NAR) contact at each hospital to gather information about how many of their surgeons who regularly operated TKAs in 2020. The hospitals not responding were consecutively contacted by phone. Two hospitals did not respond to our inquiry. Based on this, we estimated that 257 orthopedic surgeons operated TKAs in Norway at the time of our data collection, January- March 2020. The surgeons responding are not identified.

We received 128 responses between 30^th^ January—26^th^ March 2020. Responders were excluded if 1) surgeons did not work at a Norwegian hospital (*n* = 2), 2) there was a strong indication of duplicate-response (the same surgeon responding twice) (*n* = 4), 3) they responded according to a non-primary TKA (*n* = 1). The selection resulted in 121 individual responses. Single answers to questions were excluded if they 1) did not provide an answer to the question 2) were obvious printing error. By these criteria, 6 answers were excluded. 22 answers were missing from the dataset.

To assess variation within a hospital we required that the response rate from the surgeons at that hospital was at least 50% and that there were 2 or more responses.

### Techniques of good, intermediate and low level of evidence

**Fig. 1:**
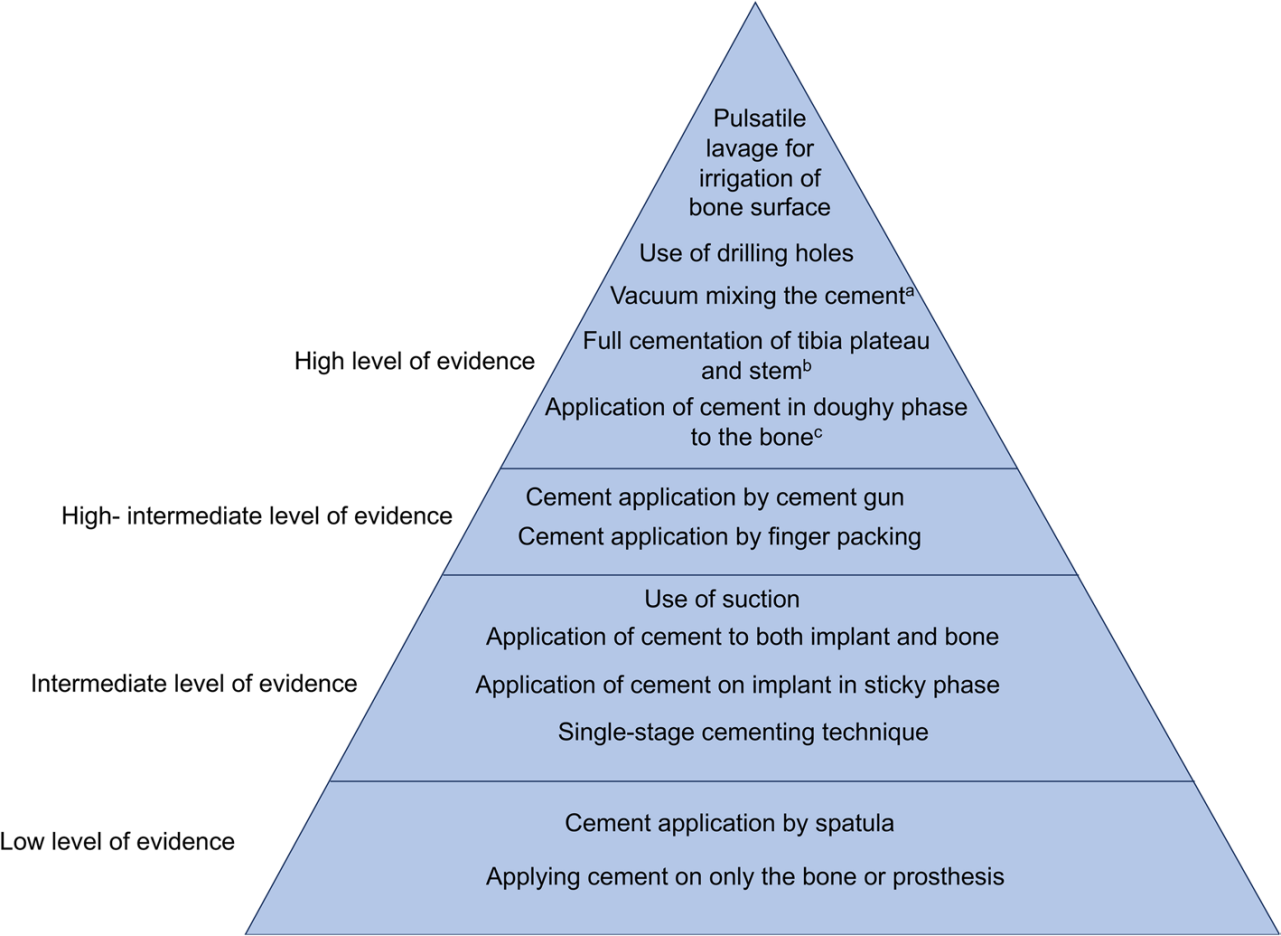
Surgical techniques classified into levels of evidence based on Refsum and Nguyens findings. **a** Vacuum mixing was not discussed by Refsum and Nguyen. **b** Cementing both the plateau and stem of the tibial component. **c** Question not asked in questionnaire

Based on the scoping review by Refsum and Nguyen [9] we classified cementing techniques into techniques with high, intermediate, and low level of evidence (Fig. 1, see Supplementary data). Vacuum mixing is also considered as a technique with high level of evidence but was not discussed by Refsum and colleagues.

The responses from the questionnaires in the Google forms were re-organized and analyzed in Microsoft excel with descriptive statistical methods, calculating frequencies in percentages.

## Results

### Questionnaire data

We obtained 121 responses to the questionnaire. It was estimated that 257 surgeons performed TKAs in Norway at the time of data collection, giving a response rate of 47%. The responders represented 45 out of 56 hospitals performing TKAs in Norway. In 20 hospitals more than half of the surgeons involved in TKA surgery responded. The majority of the responders (79%) reported they had at least 3 years of experience with performing TKAs.

### 1: How Norwegian surgeons cement TKA

#### Bone preparation

Tourniquet was widely used among our responders: 60% used it during the whole procedure, whilst 4% only used it during cementation. Drilling holes into both the femur and tibia was done by 50% of the surgeons, while 41% specified that holes were only made in sclerotic bone. A drill was used to make holes by 80% whilst 17% used impaction tools. All of the responders used pulsatile lavage to irrigate the bone. Almost all the responders, 98%, dried the bone with a gauze before applying cement (Table 1).

**Table 1:** Norwegian surgeons´ responses to questions regarding surgical technique in bone preparation in TKA

Surgical technique	Total (%)
Use of tourniquet (*n* = 121)	
Yes	72 (60)
No	45 (37)
Only while cementing	4 (3)
Drilling holes into both tibia and femur (*n* = 121)	
Yes	61 (50)
No	2 (2)
Only into sclerotic bone	50 (41)
Others ^a^	
Hybrid implants	3 (2)
Unclear ^b^	5 (4)
Device for making holes (*n* = 119)	
Drill	95 (80)
Free text answers:	
Impaction tool	20 (17)
Both drill and impaction tool	2 (2)
Missing answers	2 (2)
Impaction tools (*n* = 20):	
Awl	8 (40)
Punch	7 (35)
Ewald ^c^	2 (10)
Impactor	3 (15)
Hole diameter (*n* = 119)	
≤ 2 mm	33(28)
2.5–3.2 mm	53(45)
≥ 3.5 mm	15(13)
Missing answers	18(15)
Pulsatile lavage to irrigate bone	121 (100)
Drying bone with gauze	118(98)

Values are frequencies (%)

^a^The responders used free-text to answer

^b^The responders answered “no” and also stated hole instruments

^c^Surgical forceps

#### Cement properties and application

Applying cement to the implant in a sticky phase (before working phase) was common practice (94%). A cement gun was used for cement application by 94%. Application of cement to both the plateau and stem of the tibial component, referred as full cementation, was done by 61% of the responders. Cement was applied to both the implant and bone by 70%. Different techniques to improve cement penetration was utilized by 86%. The most utilized equipment to pressurize cement into cancellous bone was a cement gun (Table 2).

**Table 2:** Norwegian surgeons´ responses in 2020 to questions regarding cementation technique in TKA. Values are frequency (%)

Surgical technique	Total
Cement storage (*n* = 121)	
Room temperature	76 (63)
Fridge	44 (36)
Missing answers	1 (1)
Cement extraction from fridge (*n* = 44)	
At the beginning of the operation	26 (59)
Just before cementing	18 (41)
Mixing under vacuum conditions	120 (99)
Cement application on implant before working phase	114 (94)
Application method	
Cement gun	113(93)
Moistened glove	2 (2)
Others ^a^	
Both cement gun and hands	6 (5)
Cement thickness	
< 3mm	73 (60)
3–5 mm	43 (36)
> 5 mm	1 (1)
Excluded answers	4 (3)
Cementing tibial component	
Full cementation^b^	74 (61)
Surface cementation	41 (34)
Others	
Intermediate^c^	4 (3)
Surface for MB, full for AP^d^	2 (2)
Application sites	
Both bone and prosthesis (*n* = 85)	
First on prosthesis	73 (85)
First on bone	12 (14)
Missing answers	1 (1)
Only prosthesis or bone (*n* = 36)	
Only prosthesis	35 (97)
Only bone	1 (3)
Cement penetration technique	
Cement gun	72 (59)
Suction with syringe	3 (2)
Others	
Compression	9 (7)
Hammering	6 (5)
Hand	8 (7)
Spatula	1 (1)
Chisel	2 (2)
Combination of multiple tools	1 (1)
Thick layer of cement	1 (1)
No technique to enhance penetration	17 (14)
Excluded answers	2 (2)

^a^The responders used free-text to answer. Applicable for all “Others” options

^b^Full cementation: cement on both plateau and stem

^c^Intermediate: cement on the plateau and partially along the stem

^d^*MB* Metal Backed, *AP* All-poly

#### Implant insertion and cement curing

Cementation of both components in one stage was practiced by 94% of the responders. Flexing the joint to remove excess cement before full curing was done by 82%. Among these, 19% flexed the joint at least twice. A fully extended curing position was held by 57%. Only 10% of the responders used patellar resurfacing routinely for their TKAs. They all cemented the patella component in the same stage as the tibia- and femoral components and used a clamp to pressurize the component (Table 3).

**Table 3:** Norwegian surgeons´ responses in 2020 to questions regarding implant-insertion and leg-positioning during curing

Surgical technique	Total
Insertion of implant	
Single- stage	114 (94)
Two-stage	7 (6)
Cementing with trial polyethylene insert	
Yes	48 (40)
No	73(60)
Joint flexion to remove extruded cement	
No	22 (18)
Yes	99 (82)
Number of joint flexions (*n* = 99)	
Once	80 (81)
Twice or more	19 (19)
Curing knee in a fully extended position	69 (57)
Curing knee in > 45 degrees of flexion	8 (7)
Curing knee in < 45 degrees of flexion	44(36)
Holding position with your own hands	61 (50)
Patella component	12 (10)
on indication ^a^	6 (5)
Techniques for cementation of patella (*n* = 12)	
Cemented in the same stage as femur and tibia	12 (100)
Use of patellar clamp	12 (100)

Values are frequency (%)

^a)^ The responders stated under comments that patella components were cemented on indication

### 2: Comparing surgeons’ techniques to evidence-based techniques from the literature

The majority (> 90%), of the responders used cementing techniques with high level of evidence, such as irrigating the bone with pulsatile lavage and applying cement with a cement gun. However, only 61% of the responders used a full cementation technique for cementing the tibial component. When comparing surgical practice to techniques with intermediate level of evidence, 70% of the surgeons applied cement to both the implant and bone.

None of the surgeons used a spatula to apply the cement. However, 30% only applied cement to either the bone or implant (Table 4).

**Table 4:** Comparing cementing techniques used by Norwegian surgeons in 2020 to level of evidence. Values are frequency (%)

Surgical techniques	Surgeons´ practices
High level of evidence	
Irrigation of bone surface with pulsatile lavage	121 (100)
Drilling holes into bone	111 (91)^a^
Full cementation^b^	74 (61)
High-intermediate level of evidence	
Cement gun for cement application	113 (93)
Finger packing for cement application	2 (2)
Intermediate level of evidence	
Use of suction	3 (2)
Application of cement in sticky phase on implant	114 (94)
Application of cement to both implant and bone	85 (70)
Single stage cementing technique	114 (94)
Low level of evidence	
Spatula for cement application	0 (0)
Only applying cement to either bone or implant	36 (30)

Values are frequencies (%)

^a^Surgeons drilling routinely and in sclerotic bone only summed

^b^For MB components

### 3: Use of guidelines in cementing technique

We considered 20 hospitals, constituting responses from 79 surgeons, when assessing use of guidelines in cementing technique. We asked if the department followed guidelines for cementation techniques. There was disagreement between surgeons in 11 of the 20 hospitals, whilst 7 hospitals agreed upon using guidelines, and 2 hospitals agreed upon not using guidelines.

## Discussion

The purpose of this study was to compare the surgical practice among Norwegian surgeons performing TKA to recommended techniques reported in the literature. We found that for bone preparation, over 90% of the responders used techniques with high level of evidence, such as rinsing the bone using pulsatile lavage and drilling holes into the bone. Full cementation of the tibial component was done by 61% of our responders. One-stage cementation was favored among the surgeons.

A secondary aim was to investigate variation between surgeons within the same hospitals. More than half of the hospitals disagreed internally whether they had guidelines for TKA cementing technique or not.

### Pulsatile lavage

All of our responders used pulsatile lavage to clean the bone during TKA. Several studies favor pulsatile lavage to the alternatives. Both Schlegel et al. [12] and Clarius et al. [13] found a significantly higher cement penetration when the bone was rinsed with pulsatile lavage, opposed to when irrigated with syringe lavage. Recommendations from the scientific literature about bone irrigation were well implemented among our responders.

### Drilling holes

Drilling holes into the bone was common practice among our responders, and this technique has been shown to improve cement penetration into the bone. An experimental study on irrigated cadaveric bone found that drilling holes into cortical bone increased the shear and tensile strength in then bone-cement interphase significantly, compared to no holes [14]. This suggests that drilling holes can enhance fixation, especially in sclerotic bone, where cement does not penetrate as easily as in trabecular bone stock.

Ahn and colleagues [15] compared the diameter of drilling holes into sclerotic tibiae with respect to cement penetration and radiolucent lines (RLLs) in a comparative, retrospective study. They found that a larger hole-diameter of 4.5 mm increased cement penetration and had reduced the appearance of RLLs, which is associated with poor results, compared to smaller hole-diameters at 2.0 mm. Their findings suggest that choosing a wider drill enhances cement penetration into sclerotic bone. Specific diameter and depth of holes for optimal results should be assessed further.

### Surface vs full cementation of the tibial component

Among our responders, 61% did a full cementation of the tibial component. In addition, the majority of components used were metal backed according to NARs annual report from 2020. The necessity of cementing both the plateau and stem of the tibial component is a controversial topic. The rationale behind cementing the stem is to improve fixation and stability of the tibial tray. Experimental studies with composite bone have shown that full cementation was superior with regards to initial stability and micromotion of the tibial baseplate [16]. However, in a clinical trial, no difference was found between the techniques with respect to implant rotation, migration, clinical outcome [17] or long-term survival [18]. This indicates that extrapolating findings from experimental studies to clinical conditions may be problematic for this situation. Hyldahl et al. found, in their randomized trial (RCT) using radiostereometric analysis (RSA), that surface cementation of metal backed tibial components was insufficient with respect to rotation and liftoff of the implant, but sufficient when all poly components of the AGC knee was used [19].

The necessary cementing technique may vary with implant design. Hyldahl and colleagues found surface cementing to be insufficient for metal backed components and used the AGC knees in their study, while Saari and colleagues found no difference between the methods using the tibial metal backed PROFIX knee in their study. Apart from Hyldahls´ and Saaris´ papers, there are no RCTs comparing the two techniques [17, 19, 20].

### Single-stage cementation

The practice of cementing both components in a single stage was common among our responders. Cementing a TKA in one or two stages is also an area of differing opinions among researchers. The single-stage technique decreases the operating time which could reduce the risk of infection [21]. An RCT have also shown that knees cemented in a single stage had significantly less radiolucent lines (RLL) compared to two-stage cemented knees [22]. RLLs have been associated with poor implant outcome [22]. On the contrary, cementing with a two-stage technique ensures complete curing and fixation of the components before exposing the joint to movement. Experimental, cadaveric studies indicate that flexing the joint during the curing phase has a negative impact on the binding strength of the tibial plateau, as well as increasing lipid contamination in the implant–cement interface. The authors of the latter study advocated for a two-stage cementing technique to combat the issue of fat contamination of the metal plateau [23]. Our responders favored a one-stage cementation technique, but the literature is scarce. This should be investigated further. Only 40% of the responders cemented with trial polyethylene insert. This technique makes it easier to remove excess cement behind the prosthesis during single-stage cementation. With some prosthesis designs, however, it is not possible to use a trial insert if a single-stage cementation is performed, thereby making the removal of cement more difficult. The surgeon must in these cases either accept this difficulty or perform a two-stage cementation.

### Variation

Our data implies that surgeons had differing perceptions about their hospitals use of a common guideline for cementing techniques. We found 2 out of 20 hospitals agreeing upon not using common guidelines for TKA. The divided perception of this among surgeons is surprising, as it is common to have an in-hospital guideline for procedures like TKA. Further, this opens for between-surgeon variation which may affect patient outcome. The varied perception among surgeons could stem from the lack of focus on cementing technique for TKA in scientific literature. Martin and colleagues showed that American hip and knee surgeons also demonstrated variability within the TKA cementing procedure [24] Comparing to hip arthroplasty, where there is a common understanding about the cementing technique taught through courses and investigated in scientific literature, surgeons carry on the cementing technique demonstrated by their supervisors and not necessarily with techniques supported by scientific evidence for TKA. We believe there should be an increased focus towards developing common guidelines for cementing technique regarding TKA supported by findings from scientific literature.

### Limitations

This study had some limitations. Only 47% of the surgeons responded to the questionnaire. The shift towards using evidence-based techniques could be due to non-responder bias, raising the question of how representative the studied population is for all Norwegian orthopedic surgeons. The low number of responders also weakens the reliability of our results when investigating differences within hospitals. We attempted to mitigate this effect by only studying hospitals where at least 50% of TKA-operating staff responded.

The dataset had missing datapoints: there were 22 missing answers, 18 of which were drill-diameter. We excluded 6 answers from single questions. Questions about common guidelines and cement-thickness might have been unclearly expressed, and thus difficult to interpret for the responders. We used an illustration in the questionnaire to make it clearer. For use of patella components, our results would probably be different if an “on indication” option was presented to the responders, as it was pointed out by some of our responders as being a situation of discretion for surgeons.

### Future research

There has been too little focus on cementing techniques for TKA. The number of randomized clinical studies on the subject is low [17, 19, 20, 22]. Registration of cementing techniques in registries would help assess long-term effects of clinically used techniques. Currently, only the Dutch arthroplasty register systematically register data on cementing technique. Including this information also in the NAR and other arthroplasty registries may enable future register studies on the importance of cementing technique. Further, we need more laboratory studies and RCTs with accurate measures such as RSA to effectively assess the quality of cementing techniques.

## Conclusions

Based on our questionnaire-retrieved data, we found that the responders mainly used techniques of high or intermediate evidence compared to recommendations. Surgeons seem to disagree whether they use common guidelines within their hospital or not. The majority of the responders used recommended techniques from the literature when cementing TKA. Certain techniques can be considered unfortunate, such as excessive movement of the knee before the cement has cured completely. The focus on cementation techniques in TKA should be raised to develop guidelines for best practices, to mitigate the effect of unfortunate cementing technique on implant survival.

### Supplementary Information

**Additional file 1: Supplementary table 1. **Questionnaire regarding hospital affiliation, years of experience, use of common guidelines and TKA cementing techniques.

## Data Availability

The datasets used and/or analyzed during the current study are available from the corresponding author on reasonable request.

## Abbreviations

TKA: Total knee arthroplasty
NAR: Norwegian arthroplasty register
FC: Full cementing
MB: Metal- backed
AP: All poly
RLL: Radio lucent lines
RCT: Randomized controlled trial
RSA: Radiostereometric analysis
AGC: Anatomic graduated component
