# Supplementary material for: Cementing techniques for total knee arthroplasty in Norwegian hospitals; a questionnaire-based study
Source: BMC Musculoskelet Disord. 2023 Nov 18;24:900. doi: 10.1186/s12891-023-07040-2 (PMC10656824; doi:10.1186/s12891-023-07040-2)
Supplement: Supplementary file 1 — Additional file 1: Supplementary table 1. Questionnaire regarding hospital affiliation, years of experience, use of common guidelines and TKA cementing techniques. [file 12891_2023_7040_MOESM1_ESM.docx]

Original questionnaire in Norwegian: <https://docs.google.com/forms/d/1b-dH4m7VTxCXnf9SUZJpiJQ8vvOkUhxGcjc1gOLy2-Q/edit#responses>

Supplementary table 1: Questionnaire regarding hospital affiliation, years of experience, use of common guidelines and TKA cementing techniques

| Questions | Options presented to  questionnaire participants |
| --- | --- |
| Name the hospital you are employed in (no hospitals will be identified in published article) | Free text |
| Do you have more than 3 years of experience with total knee arthroplasty (TKA) surgery | Yes  No |
| Do the surgeons at your hospital utilize a common guideline for cementing technique regarding TKA? | Yes  No  I don´t know |
| If you use a common guideline, how long has this been utilized? State number of years. | Free text |
| Do you utilize tourniquet during the operation? | Yes  No  Only during cementation |
| If you use tourniquet, what is your time limit for tourniquet use? State number of minutes. | Free text |
| Are holes drilled in both tibia and femur? | Yes  No  Only in sclerotic bone |
| If you make holes into the tibia and femur, what tool is used to make the holes? | Drill  Peang  Others (free text) |
| If you use a drill to make holes, what drill size do you use? State in millimeters. | Free text |
| Where is the cement stored? | In fridge  In room temperature |
| If the cement is stored in the fridge, when is the cement taken out from the fridge? | In the beginning of the operation  Just before mixing the cement |
| Do you use a vacuum mixing system to mix the cement? | Yes  No |
| Do you use pulsatile lavage on the bone? | Yes  No |
| Do you rinse the bone with other methods than pulsatile lavage? | Free text |
| Do you dry the bone with a gauze before cementing? | Yes  No |
| Is the cement applied to the implant while it is sticky, meaning before the working phase? | Yes  No |
| How do you apply the cement? | Cementing gun  Finger packing  Other (free text) |
| When finger packing, do you wet the glove before cementing? | Yes  No |
| Which parts of the tibia component is cemented? | The whole component (stem + tibial plateau)  Just the tibial plateau  Others (free text) |
| When cementing the components, is cement applied to both the bone and implant? | Yes  No |
| If you only apply cement to either the implant or the bone, is cement only applied to the implant or only to the bone? | Only implant  Only bone |
| If you apply cement to both the implant and the bone, is the cement applied to the bone or implant first? | First bone  First implant |
| Do you use any technique to pressurize cement into the bone? | Cementing gun pressuring cement into the bone  Suction  Other (free text) |
| How thick cement mantel do you strive to achieve? State in millimeters from the edge of the implant to the metaphyseal bone as shown on the illustration.  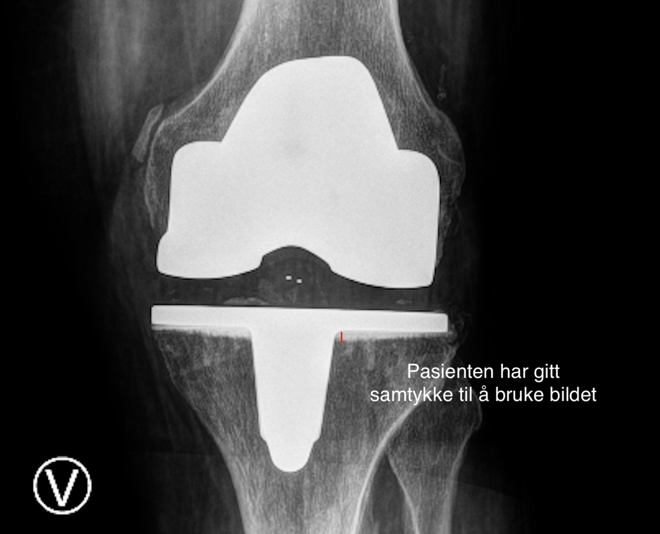 | Free text |
| When the implant is inserted, is this done in one (femur and tibia together) or two stages? | One stage  Two stages |
| Do you cement with a trial polyethylene insert? | Yes  No |
| After inserting the components, how many times do you extend and flex the knee joint to remove excess cement? | 0 times  1 time  2 times or more |
| Is the bone held in a fully extended position with constant compression during the curing phase? | Yes  No |
| If you don’t hold a fully extended position, which position of flexion is the leg held in whilst curing? State in number of degrees. | Free text |
| Do you hold the leg with your own hands until the cement is fully cured? | Yes  No |
| Do you routinely use a patella component in TKA? | Yes  No |
| If you routinely use a patella component, when is the patella component cemented? | Before the femoral- and tibial component  Together with the femoral and tibial component  After the femoral and tibial component |
| If you use a patella component, do you use a clamp to keep the patella still? | Yes  No |
| If you have any comments regarding other relevant factors that have not been mentioned in this questionnaire you may state them in the section below. | Free text |
